# Dysregulated autophagy in muscle precursor cells from humans with type 2 diabetes

**DOI:** 10.1038/s41598-019-44535-2

**Published:** 2019-06-03

**Authors:** T. I. Henriksen, L. V. Wigge, J. Nielsen, B. K. Pedersen, M. Sandri, C. Scheele

**Affiliations:** 1 The Centre of Inflammation and Metabolism and the Centre for Physical Activity Research, Rigshospitalet, University of Copenhagen, Copenhagen, Denmark; 2 Department of Biology and Biological Engineering, Chalmers University of Technology, 41296 Gothenburg, Sweden; 3 Venetian Institute of Molecular Medicine, via Orus 2, 35129 Padova, Italy; 4 Novo Nordisk Foundation Center, Section for Basic Metabolic Research, Faculty of Health and Medical Science, University of Copenhagen, Copenhagen, Denmark

**Keywords:** Macroautophagy, Muscle stem cells, Type 2 diabetes

## Abstract

Autophagy is active during cellular remodeling including muscle differentiation. Muscle differentiation is dysregulated in type 2 diabetes and we therefore hypothesize that muscle precursor cells from people with type 2 diabetes (T2DM) have a dysregulation of their autophagy leading to impaired myogenesis. Muscle precursor cells were isolated from people with T2DM or healthy controls and differentiated *in vitro*. Autophagy marker levels were assessed by immunoblotting. Differentially expressed autophagy-related genes between healthy and T2DM groups were identified based on a previously published RNA-sequencing data-set, which we verified by RT-qPCR. siRNA was used to assess the function of differentially expressed autophagy genes. Basal autophagy increases during human muscle differentiation, while T2DM muscle cells have reduced levels of autophagy marker ATG7 and show a blunted response to starvation. Moreover, we demonstrate that the 3 non-canonical autophagy genes *DRAM1*, *VAMP8* and *TP53INP1* as differentially expressed between healthy and T2DM groups during myoblast differentiation, and that *T53INP1* knock-down alters expression of both pro-and anti-apoptotic genes. *In vitro* differentiated T2DM muscle cells show differential expression of autophagy-related genes. These genes do not regulate myogenic transcription factors but may rather be involved in p53-associated myoblast apoptosis during early myogenesis.

## Introduction

Skeletal muscle function depends on the continuous repair and maintenance of muscle fibers in a complex process relying on satellite cells: a population of muscle precursor cells residing beneath the basal lamina, with the capacity to expand and progress through the myogenic program upon activation^1,2^. Throughout differentiation of these muscle precursor cells a considerable reprogramming of gene expression occurs; likewise, a substantial remodeling of cellular structures takes place^3,4^.

A candidate mechanism behind this remodeling is autophagy; a major cellular degradation pathway in which intracellular organelles can be broken down and recycled, thus allowing for cellular clean-up and homeostasis^5^. Adaptation to cellular stress is a key function of autophagy, and autophagy has concomitantly been shown to protect against metabolic diseases such as insulin resistance and diabetes^6^. Beyond its roles as a stress and starvation response, autophagy is also involved in cell survival, differentiation and development^7,8^, and importantly, recent murine-derived cell-based studies have shown that autophagy is required for *in vitro* myogenesis^4,9^. Moreover, it is becoming increasingly clear that autophagy is required for maintenance of skeletal muscle tissue. This is illustrated by several murine knock-out model-studies of autophagy-associated proteins showing that autophagy is critical for maintaining skeletal muscle mass, as well as muscle metabolism and function^10–13^, thus underlining the importance of functional autophagy in skeletal muscle.

Skeletal muscle tissue is a main target of insulin-mediated glucose uptake, and skeletal muscle insulin resistance is a central step in type 2 diabetes disease progression^14^. Considering that autophagy is under direct regulation by nutrient availability^15^ and the wide-ranging metabolic perturbations in T2DM, surprisingly few studies have investigated how type 2 diabetes affects skeletal muscle autophagy. The IGF-I/ PI3K/AKT signaling cascade directly inhibits skeletal muscle autophagy^16^, and as this pathway is dysregulated in skeletal muscle from T2DM individuals^17^, it is plausible that dysfunctional insulin signaling may lead to altered autophagy levels in skeletal muscle in type 2 diabetes. While a previous study found that both basal and insulin-stimulated autophagy levels were largely unaltered in skeletal muscle biopsies from patients with type 2 diabetes^18^, this has not yet been investigated in isolated muscle precursor cells during differentiation. Given the dynamic nature of autophagy it is possible that potential differences are blunted at tissue level, yet important at a cellular level. We have recently shown that differentiation is impaired in muscle precursor cells isolated from T2DM donors^19^. In the present study, our primary objective was therefore to investigate if muscle precursor cells isolated from type 2 diabetic donors would have altered levels of autophagy markers, suggesting altered autophagy, compared with cells from healthy control donors. Furthermore, we wanted to explore the role of autophagy in the T2DM-mediated dysregulated myogenesis.

## Materials and Methods

### Human muscle precursor cell donors

Muscle precursor cells were obtained from a subset of male healthy (n = 5) or type 2 diabetic (n = 5) donors (Table 1) included in a previously described study^20^. RNA-seq data was obtained from a previous study with a partial overlap in cell culture donors with the present study^21^. The WHO diagnostic criteria for type 2 diabetes were used as basis for inclusion^22^. All subjects gave written informed consent prior to commencement of study, which was performed according to the Declaration of Helsinki and approved by The Regional Committee on Biomedical Research Ethics in Denmark (KF 01-141/04).

**Table 1:** Clinical characteristics of muscle precursor cell donors.

	Healthy (n = 5)	T2DM (n = 5)
Sex	5 M	5 M
Age (years)	50–59	52–66
BMI	23.8 ± 1.3	27.4 ± 0.9**
Fasting glucose	5.1 ± 0.6	9.4 ± 3.4*
OGTT 2-hour glucose	5.5 ± 0.8	17.9 ± 6.3**
Fasting insulin	42.2 ± 11	61.4 ± 17
OGTT 2-hour insulin	305.8 ± 197	223.0 ± 161
HOMA-IR	1.6 ± 0.5	4.3 ± 1.9*
VO_2_ max (L/min)	2.9 ± 0.7	2.43 ± 0.7
VO_2_ max (mL/min/kg)	35.9 ± 9.9	26.8 ± 6.4
**Donors for cells used for RNA sequencing in** ^21^		
	**Healthy (n** = **6)**	**T2DM (n** = **6)**
Sex	3 M/3 F	3 M/3 F
Age (years)	48–63	50–63
BMI	24.0 ± 0.6	24.4 ± 2.7
Fasting glucose	5.4 ± 0.6	9.0 ± 3.8*
OGTT 2-hour glucose	5.2 ± 1.0	18.0 ± 7.3**
Fasting insulin	25 ± 7	38 ± 35
OGTT 2-hour insulin	175 ± 123	281 ± 303
HOMA-IR	0.47 ± 0.13	0.81 ± 0.72
VO_2_ max (L/min)	2.4 ± 0.8	1.63 ± 0.4
VO_2_ max (mL/min/kg)	32.7 ± 8.8	23.2 ± 3.26

Ages are shown as ranges; all other data are means ± SE. BMI, body mass index (kg/m^2^); OGTT, oral glucose tolerance test. Glucose values are mmol/L, insulin values are pmol/L. *P < 0.05, **P < 0.001, ****P < 0.0001.

### Cell culture

Satellite cells were obtained from *vastus lateralis* muscle biopsies as previously described^23^. Fat and visible connective tissue was removed, and the muscle biopsy was minced into small pieces and digested in buffer containing 0.05% trypsin-EDTA, 1 mg/ml collagenase IV and 10 mg/ml BSA for 5 min at 37 °C. Digestion solution containing released muscle precursor cells was then transferred to cold FBS for trypsin inactivation. The solution was centrifuged at 800 g for 7 min. The supernatant was removed and washed with F10 nutrient mixture (HAM) medium. To minimize fibroblast contamination, the cell suspension was pre-plated in a culture plate for 3 hours in growth medium containing 20% FBS, 1% penicillin/streptomycin (PS) and 1% Fungizone antimycotic (FZ) in F10/HAM. The unattached muscle precursor cells were then seeded onto culture flasks coated with Matrigel (0.01% Matrigel in F10/HAM + supplemented with 1% PS) and cultured for 4 days in growth medium in a humidified incubator with 5% O_2_ and 5% CO_2_ at 37 °C. Cell culture medium was changed after 4 days of incubation and then after every second day. At 100% confluency, cells were transferred to intermediate medium (Dulbecco’s modified Eagle’s medium (DMEM) containing 1 g/L glucose, 10% FBS and 1% PS) to induce alignment of muscle precursors. After 2 days, medium was changed into differentiation media (DMEM containing 4.5 g/L glucose, 2% horse serum (HS) and 1% PS) to induce differentiation into myotubes (myocytes). All cells were tested negative for mycoplasma contamination. F10/ HAM, HBSS, DMEM, FBS, HS, PS and FZ were obtained from Invitrogen (Taastrup, Denmark). Bafilomycin A1 was from Invivogen (Toulouse, France). Experiments were performed on cells at passages 5 to 6.

### Immunomagnetic sorting of CD56+ precursor cells

To reduce the presence of non-muscle cell types all cell cultures were sorted using CD56-conjugated magnetic microbeads (Miltenyi Biotec, Lund, Sweden). Cells were grown until ~50% confluent and incubated with CD56 primary antibody conjugated microbeads, and subsequently isolated by positive selection according to the manufacturer’s instructions.

### siRNA transfection

Cells were transiently transfected with small interfering RNA (siRNA) oligo pools specifically targeting *DRAM1*, *VAMP8* and *TP53INP1* mRNA respectively, or a non-targeting scrambled siRNA pool (On-Target Plus, Dharmacon, Søborg, Denmark). Transfections were performed in >90% confluent, undifferentiated cells using a final concentration of 20 nM siRNA with Lipofectamine RNAimax (Invitrogen). A Lipofectamine-only control was included as well; all statistics for siRNA experiments were performed comparing the effects of siRNA with the effects of the scrambled control. The Lipofectamine control did not differ from the scrambled siRNA control. Transfections were performed in DMEM containing 10% FBS and no antibiotics, and subsequently incubated for 24 hours, at which point medium was changed to DMEM containing 10% FBS and 1% PS. Cells were harvested at either 48 hours (Day 0) or 7 days (Day 5) after siRNA transfection.

### Immunoblot analysis

Cells were washed in ice-cold PBS and lysed using lysis buffer (20 mM Tris, pH 7.5, 150 mM NaCl, 1 mM EGTA, 1 mM EDTA, 0.1% Triton X-100, protease inhibitor ((1 tablet/10 ml; Boehringer-Roche Diagnostics, Copenhagen, Denmark), 1% phosphatase inhibitor cocktail (Sigma-Aldrich, Brøndby, Denmark)). Cell lysates were centrifuged at 12000 g, 4 °C for 5 min and supernatants were collected. Protein concentration was determined by the Bradford reagent. 10 µg cell lysates were subjected to SDS-PAGE on 4–15% precast gels (Biorad), transferred to polyvinylidene difluoride (PVDF) membranes and immunoblotted with primary antibodies (1:1000) as indicated in the figure legends. Primary antibodies were detected using species-appropriate horseradish peroxidase-conjugated secondary IgG antibody. Protein signals were visualized using FEMTO enhanced chemiluminescence and Biorad Chemidoc XRS imager. The signal bands were quantified using Image J software (NIH, Bethesda, MD, http://rsb.info.nih.gov/ij). Anti-ULK1, ATG7, and ATG5 antibodies were from Cell Signaling (MA, US) and anti-LC3 antibody was from Nanotools (Teningen, Germany).

### Immunoflourescence microscopy

*In vitro* differentiated human muscle stem cells were fixated with 4% Formaldehyde (Sigma) and then permeabilized with 0.5% Triton X-100. Cells were then incubated with ActinGreen 488 ReadyProbes and a nuclear counterstain was performed with Nucblue Fixed Cell stain ReadyProbes (Molecular probes). Fluorescence microscopy was performed with an EVOS FL (Thermo Fisher).

### RNA isolation and quantitative real-time PCR

Total RNA was extracted from cells using TRIzol (Invitrogen, Taastrup, DK) according to manufacturer’s instructions. Total RNA was dissolved in RNase-free water and quantified using a Nanodrop ND 1000 (Saveen Biotech ApS, Arhus, Denmark). 0.2 µg of total RNA was reverse transcribed using the High Capacity Reverse Transcription kit (Applied Biosystems, Foster City, CA) according to manufacturer’s protocol. Real-time quantitative PCR was performed in triplicate using an ABI-PRISM 7900 (Applied Biosystems). Primer sequences are listed in Table 2; primer targets and genomic locations are listed in Table S1. Data analysis was performed using the comparative method (ΔΔCT).

**Table 2:** Primer sequences for RT-qPCR.

Gene	Ensembl ID	Forward primer	Reverse primer
*B2M*	*ENSG00000166710*	GAGTATGCCTGCCGTGTGAA	TTCAAACCTCCATGATGCTGC
*BAD*	*ENSG00000002330*	AGACCCGGCAGACAGATGAG	AGGAAGTCCCTTCTTAAAGGAGTC
*BAX*	*ENSG00000087088*	ATGTTTTCTGACGGCAACTTC	ATCAGTTCCGGCACCTTG
*BCLX*	*ENSG00000171552*	AAAAGATCTTCCGGGGGCTG	TCTGAAGGGAGAGAAAGAGATTCA
*DRAM1*	*ENSG00000136048*	GTCAACCCCTTCCTCCCGTA	TCGTGGCTGCACCAAGAAAT
*MYH2*	*ENSG00000125414*	TGTCTCACTCCCAGGCTACA	CCAAAAACAGCCAATTCTGAG
*MYOD*	*ENSG00000129152*	CACTACAGCGGCGACTCC	TAGGCGCCTTCGTAGCAG
*MYOG*	*ENSG00000122180*	GCTCAGCTCCCTCAACCA	GCTGTGAGAGCTGCATTCG
*P21*	*ENSG00000124762*	TCACTGTCTTGTACCCTTGTGC	GGCGTTTGGAGTGGTAGA
*P53*	*ENSG00000141510*	AGGCCTTGGAACTCAAGGAT	CCCTTTTTGGACTTCAGGTG
*PAX7*	*ENSG00000009709*	CGCCCATTGATGAAGACC	CGGGATTCCCTTTGGAAG
*PPIA*	*ENSG00000196262*	ACGCCACCGCCGAGGAAAAC	TGCAAACAGCTCAAAGGAGACGC
*RB1*	*ENSG00000139687*	GGATCAGATGAAGCAGATGGA	CATTCGTGTTCGAGTAGAAGTCA
*TBP*	*ENSG00000112592*	GAACATCATGGATCAGAACAACAG	ATAGGGATTCCGGGAGTCAT
*TP53INP1*	*ENSG00000164938*	AAATGTTTGTGGGTGAAGTCAG	TGCTGAGAAACCAGTGCAAG
*VAMP8*	*ENSG00000118640*	AAGCCACATCTGAGCACTTCAA	CCAGTGGCAAAGAGCACAATG
*18* *S*	*ENSG00000225840*	Applied Biosystems assay ID Hs99999901_s1	

### Identification of target autophagy genes

GO-term gene members were acquired using the Bioconductor R package biomaRt as previously described^21^. All genes belonging to the GO-term Autophagy (GO:0006914) were cross-checked against the analysis results in^21^, and selected genes that were significantly differentially expressed (adjusted p-value < 0.05) in T2DM subjects compared to healthy controls were chosen for further investigation.

### Statistical analyses

Statistical analyses were performed using GraphPad Prism 6.0 (GraphPad Software Inc., La Jolla, CA, USA). All data within figures are presented as means ± SEM. Data in tables are presented as means ± SD. For comparisons between two groups, a Student’s t-test was used. For group differences and multiple comparisons, statistical analysis was performed using two-way ANOVA with Holm-Sidak post -hoc testing.

## Results

### Impairment in autophagy response in differentiated T2DM muscle cells

**Figure 1:**
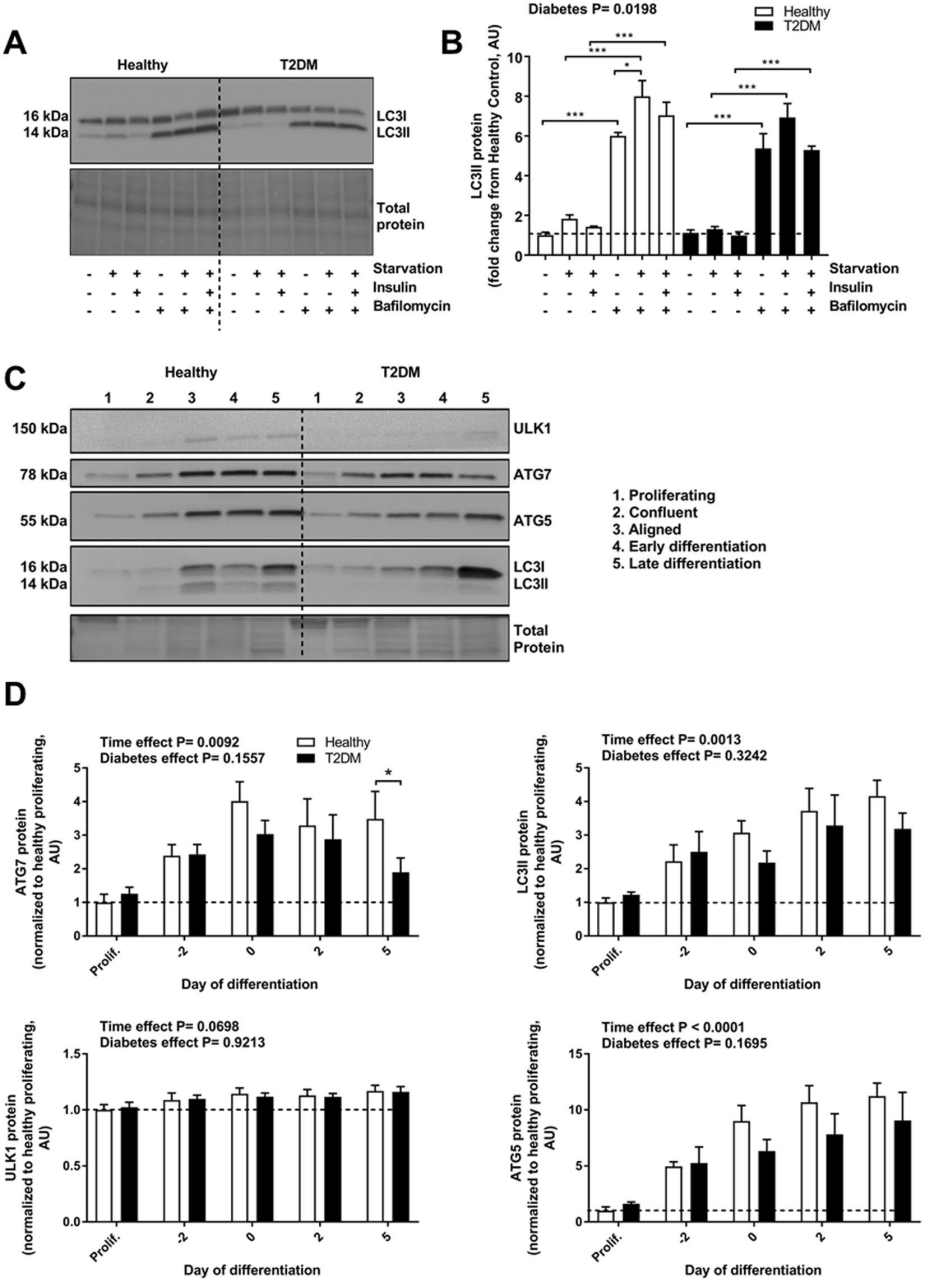
Autophagy protein expression in myoblasts from healthy and T2DM donors. (**A**,**B**) Fully differentiated myotubes from healthy control or T2DM groups were starved in HBSS medium (4 hours), and treated with 100 nM insulin (30 minutes) and/ or Bafilomycin A1 (10 nM, 4 hours). Protein levels of LC3I/II were measured by immunoblotting; representative blots are shown. (**C**) Myoblasts from healthy controls (n = 5) or T2DM donors (n = 5) were harvested under proliferation, at confluence (day -2), as aligned, undifferentiated myoblasts (day 0), at early differentiation, after 2 days in differentiation medium (day 2) and as fully differentiated myotubes, after 5 days in differentiation medium (day 5). Protein levels of ULK-1, ATG7, ATG5 and LC3I/II were measured by immunoblotting. Representative blots are shown. Two samples harvested from proliferating cells were not included in analysis, as protein levels were too low. A Reactive Brown stain was used to visualize total protein. (**D**) Quantifications of immunoblots shown in (**c**). Protein levels were normalized to proliferating healthy controls. Data are means ± SEM. * indicates P < 0.05; ***P < 0.001.

Considering the numerous metabolic defects in T2DM muscle^14^, we hypothesized that T2DM myocytes would show an impaired autophagy response when presented to a metabolic challenge. We therefore treated cells with HBSS medium for 4 hours to induce starvation, followed by re-addition of insulin to the starvation medium. Re-addition of insulin did not result in decrease of protein abundance of Microtubule-associated protein 1A/1B-light chain 3 II (LC3II) levels in any of the groups and there was no over-all difference between groups in response to this treatment (Fig. 1A,B). The effects of insulin on LC3II in muscle tissue have previously been shown to be dependent on glycemic status^18^; the lack of autophagy response to insulin in our study might therefore be due to the relatively high glucose concentration in the cell culture media. Interestingly, while both healthy and T2DM groups showed accumulation of LC3II in response to the vacuolar H^+^ ATPase-inhibitor Bafilomycin A (used to assess autophagy flux), only the healthy group showed a significant increase in LC3II when exposed to starvation in the presence of Bafilomycin A (Fig. 1B), suggesting that the dynamic activation of autophagy in response to a metabolic challenge is blunted in the T2DM group. Moreover, protein levels of LC3II were also differentially regulated between healthy and diabetic groups with an overall decrease in the T2DM cells (Fig. 1B) indicating a lower level of autophagy in this group.

Given that muscle precursor cells derived from people with T2DM have a dysregulated p53-dependent differentiation^19^, we next aimed to determine whether the disturbed regulation of autophagy was reflected by dysregulation of autophagy-associated proteins during human myoblast differentiation. To address this question, cells were grown in culture and harvested at the time points indicated in Fig. 1. Immunoblot analysis of Autophagy related 7 and 5 (ATG7 and ATG5), unc-51 like autophagy activating kinase 1 (ULK1) and LC3II revealed a marked upregulation of all four proteins as cells progressed through differentiation (Fig. 1C,D). These markers of autophagy were chosen for analysis to ensure that proteins involved in both early and later steps of the autophagic process would be assessed. At day 5, when myotubes were fully differentiated, ATG7 was markedly lower expressed in the cells derived from people with T2DM, whereas the rest of the assessed proteins did not differ between groups (Fig. 1D).

Thus, we show that basal autophagy increases during myoblast differentiation also in humans. We also found that muscle cells from T2DM donors have an blunted autophagy response and reduced levels of ATG7, a rate limiting protein of the conjugation system and therefore, of LC3 lipidation and autophagosome formation. Beside autophagy, ATG7 has been previously shown to be important for handling metabolic stress by targeting p53 activity^24^.

### *DRAM1*, *VAMP8* and *TP53INP1* are differentially expressed in T2DM muscle precursor cells

**Figure 2:**
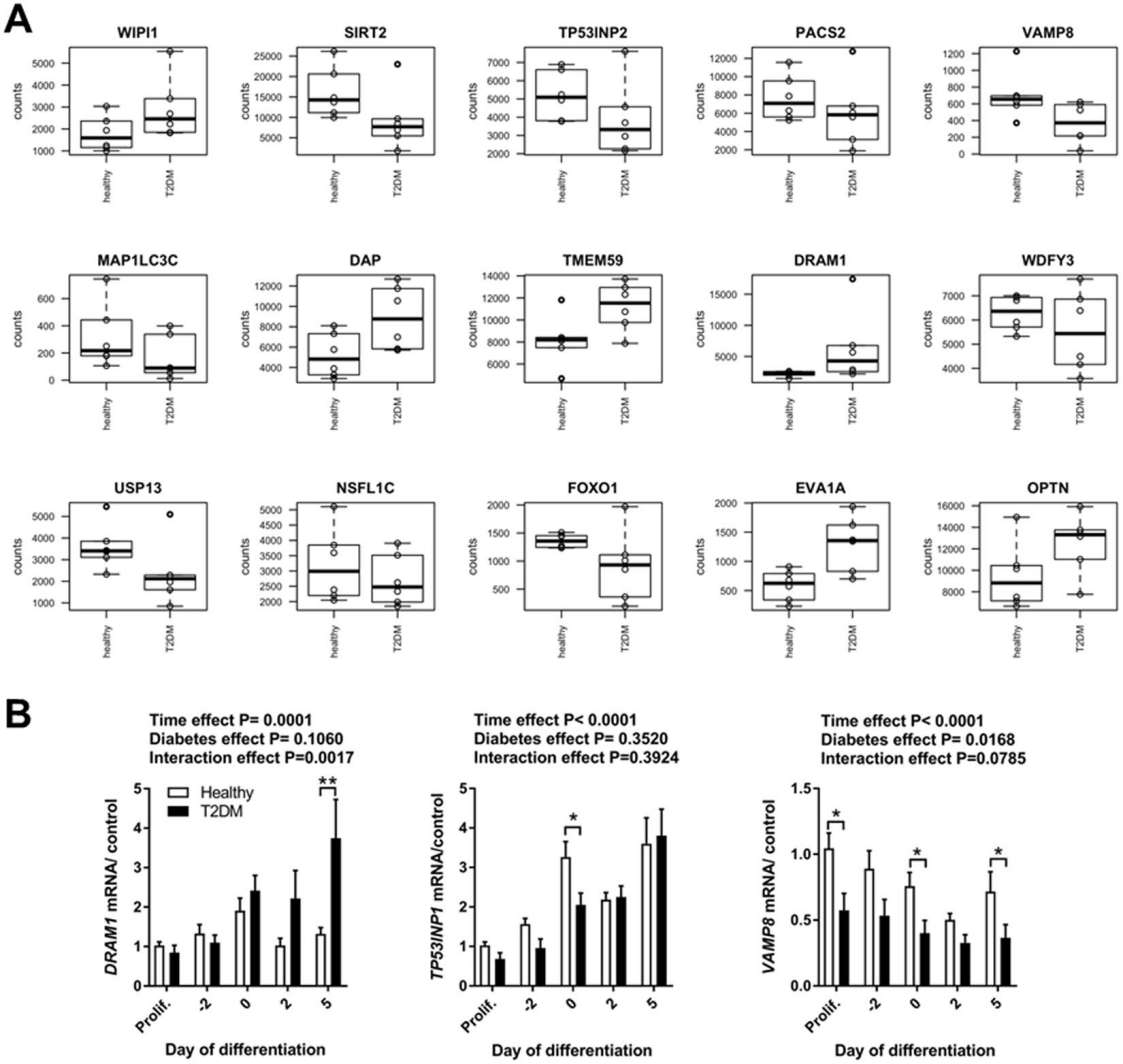
*DRAM1*, *VAMP8* and *TP53INP1* expression in myoblast derived from humans with T2DM and healthy control subjects. (**A**) Gene counts from ^21^for identified differentially expressed autophagy-related genes (adjusted p-value < 0.5) in T2DM subjects (n = 6) versus healthy controls (n = 6). (**B**) Myoblasts from healthy controls or T2DM donors were harvested under proliferation, at confluence (day -2), as aligned, undifferentiated myoblasts (day 0), at early differentiation, after 2 days in differentiation medium (day 2) and as fully differentiated moytubes, after 5 days in differentiation medium (day 5). *DRAM1*, *VAMP8* and *TP53INP1* mRNA was measured by RT-qPCR in healthy or T2DM muscle precursor cells. mRNA expression was normalized to the geometric mean of 18 *s*, *B2M* and *PPIA* mRNA. Data are means ± SEM. * indicates P < 0.05; **P < 0.01.

To further investigate the molecular dysregulations behind the reduced autophagy response to metabolic stress, we took on a global approach. In a previous study, we performed RNA sequencing to describe the transcriptional profiles of muscle cells derived from healthy or T2DM donors^21^. Based on these previous results, we identified several autophagy-related genes as differentially expressed between groups (Fig. 2A). The genes Damage-Regulated Autophagy Modulator protein 1 (*DRAM1*) and Vesicle Associated Membrane Protein 8 (*VAMP8*) were two of the most up- or down-regulated genes respectively (Fig. 2A) and were therefore chosen for further analysis. The autophagy markers analyzed by immunoblotting in Fig. 1 were not detected as differentially expressed in the RNA-seq dataset when correcting for multiple testing, nor were markers of myogenesis or apoptosis. Another differentially expressed gene, Tumor Protein 53-inducible nuclear protein 2 (*TP53INP2*) was previously shown to regulate muscle mass and autophagy and is downregulated in muscle tissue in type 2 diabetes^25^. We chose to also include the TP53INP2 homologue Tumor Protein 53-inducible nuclear protein 1 (*TP53INP1*) for analysis, as it was previously identified as a diabetes susceptibility locus^26^, yet had no described role in skeletal muscle to date.

Using qPCR analysis, we verified that *DRAM1*, *VAMP8* and *TP53INP1* were indeed differentially regulated in T2DM cells (Fig. 2B), whereas *TP53INP2* showed differential expression in proliferating cells only (Fig. S1). Only *VAMP8* was consistently downregulated over time in the T2DM group, whereas *DRAM1* was upregulated in fully differentiated T2DM muscle cells and *TP53INP1* was downregulated in T2DM muscle precursors at day 0 of differentiation (Fig. 2B). Interestingly, both DRAM1 and TP53INP1 have previously been reported to positively regulate the capacity of p53 to control cell cycle exit and induce apoptosis^27,28^. p53 also plays a role in the context of myogenesis, since activation of the p53 target gene p21is a mandatory step for muscle precursor cells to exit the cell cycle and proceed through the myogenic programme^29,30^. Since we have previously shown that muscle precursor cells from humans with type 2 diabetes have impairments in p53/p21-dependent myogenesis^19^, we therefore decided to further investigate the role of DRAM1, VAMP8 and TP53INP1 in myogenesis.

### Loss of *TP53INP1* results in enlarged myotubes

**Figure 3:**
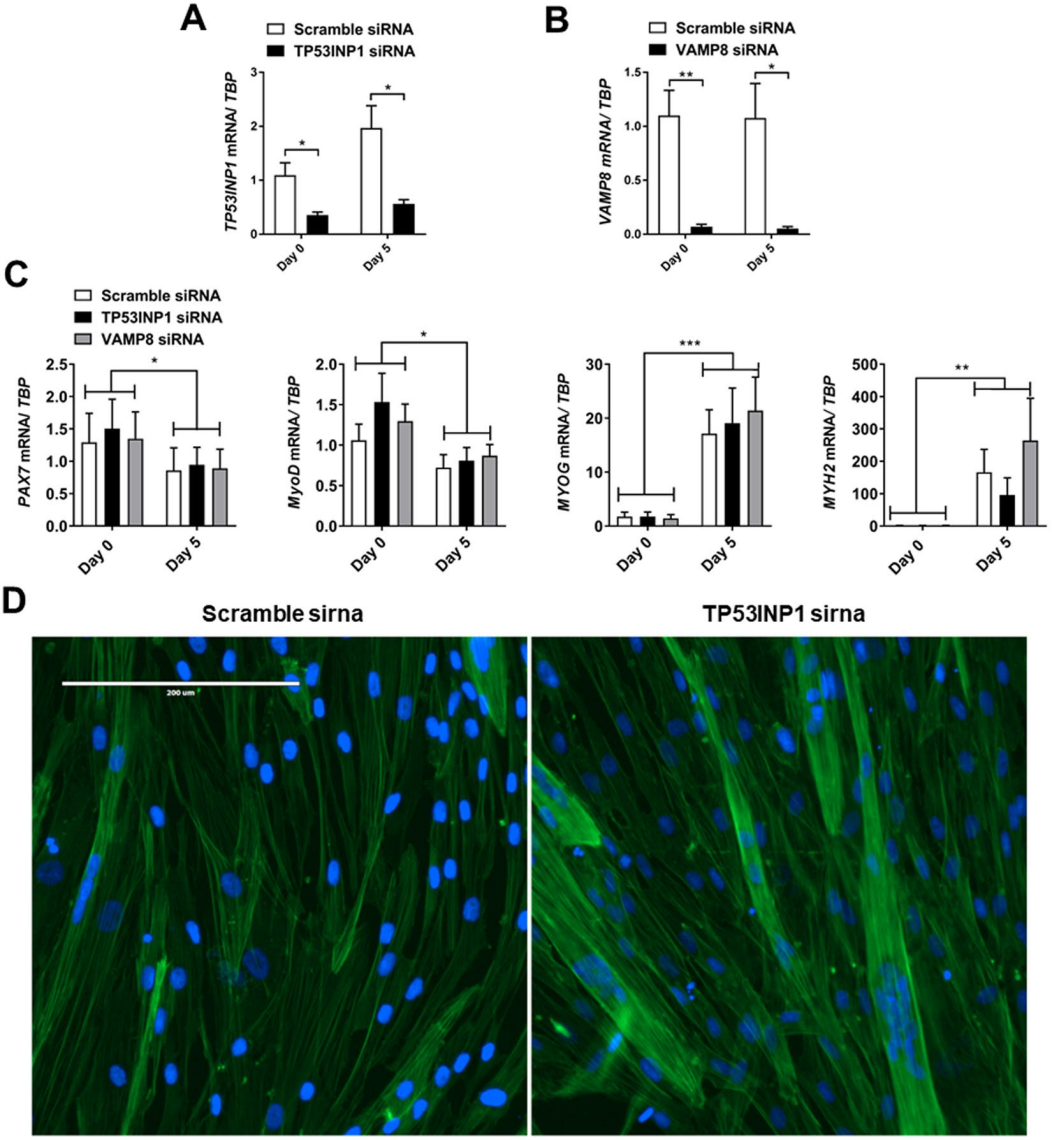
Effect of *VAMP8* and *TP53INP1* knock down on myogenic marker expression. Myoblasts from healthy controls (n = 5) were treated with 20 nM siRNA oligonucleotides targeting *VAMP8* or *TP53INP1*, or with a non-targeting scrambled control. A lipofectamine-only control was included for each experiment (data not shown) to confirm no off-target effects of the scrambled siRNA control. Cells at >90% confluence were treated with siRNA and harvested 48 hours or 7 days after siRNA treatment; indicated as day 0 or day 5 respectively. (**A**,**B**) *VAMP8* and *TP53INP1* mRNA levels were measured by RT-qPCR and normalized to *TBP* mRNA. The same control was used for both knock-downs. (**C**) Light microscopy images of siRNA-treated cells at 4X magnification; scale bar indicates 1000 µM. (**D**) Expression of myogenic markers *PAX7*, *MYOD*, *MYOG* and *MYH2* in healthy control myoblasts treated with *VAMP8*, *TP53INP1* or control siRNA. Data are means ± SEM. * indicates P < 0.05; **P < 0.01, ***P < 0.001.

In accordance with previous studies^4,9^ we found that autophagy appeared to increase during muscle cell differentiation, indicating that autophagy plays a role in this process. We therefore hypothesized if knock down of the autophagy-associated genes *DRAM1*, *VAMP8* or *TP53INP1* would have an impact on myogenesis. To assess this, cells were grown until confluent and siRNAs against *DRAM1* in T2DM cells and against *VAMP8* and *TP53INP1* in cells from healthy donors were used to knock down their respective target mRNAs. Cells were harvested 48 hours and 7 days after knockdown respectively (indicated as day 0 and day 5). mRNA levels of *DRAM1* (Fig. S3A) and *VAMP8* and *TP53INP1* (Fig. 3A,B) were significantly reduced after siRNA treatment and remained low throughout differentiation suggesting that siRNA treatment was effective. Knockdown of either *DRAM1* or *VAMP8* or *TP53INP1* did not appear to affect cell viability as assessed by light microscopy at day 0 or day 5 of differentiation, since siRNA-treated cells all proceeded to form large, multinucleated myotubes during differentiation (Figs S2, S3B). However, whereas *DRAM1* and *VAMP8* siRNA treatment did not affect myotube morphology, knock down of *TP53INP1* lead to formation of visibly larger myotubes when compared to control cells (Figs 3D; S2). We next measured the mRNA expression of myogenic markers PAX7, MyoD, myogenin and myosin heavy chain 2 a (MYH2), to see if loss of *DRAM1*, *VAMP8* or *TP53INP1* would affect their expression. However, while all myogenic markers showed significant changes over time, we did not detect any effect of *DRAM1*, *VAMP8* or *TP53INP1* knockdown on their expression (Figs 3C; S2C).

### Loss of *TP53INP1* increases pro-and antiapoptotic markers

**Figure 4:**
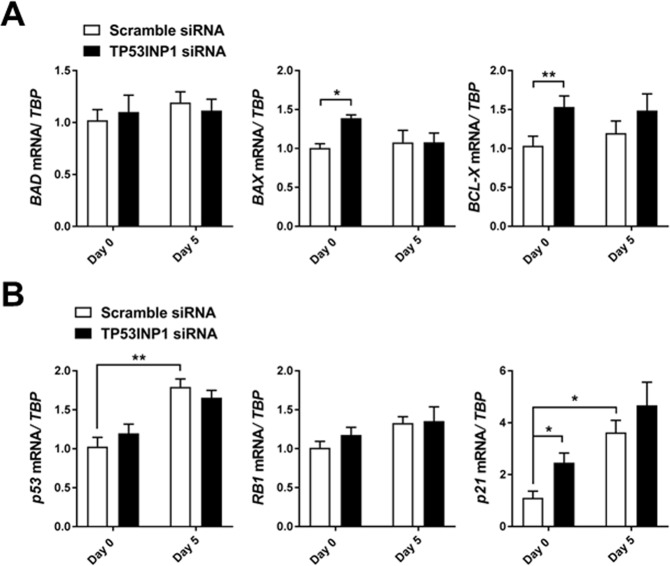
Effect of *TP53INP1* knock down on *P53* and apoptosis-related gene expression. Myoblasts from healthy controls (n = 5) were treated with 20 nM siRNA oligonucleotides targeting TP53INP1, or with a non-targeting scrambled control. A lipofectamine-only control was included (data not shown) to confirm no off-target effects of the scrambled siRNA control. Cells at >90% confluence were treated with siRNA, and harvested 48 hours or 7 days after siRNA treatment. (**A**) mRNA expression of pro-apoptotic markers *BAD* and *BAX* or anti-apoptotic marker *BCL-X*. (**B**) mRNA expression of *P53*, *MDM2*, *RB1* and *p21* in healthy control myoblasts treated with TP53INP1 or control siRNA. (**B**) Data are means ± SEM. * indicates P < 0.05; **P < 0.01, ***P < 0.001.

Growth factor withdrawal signals to myoblasts to exit the cell cycle and undergo differentiation; however, loss of growth factor signaling can also induce myoblast apoptosis^31,32^. TP53INP1 is a p53 target gene involved in the regulation of p53-mediated apoptosis^27,33,34^. We therefore hypothesized that loss of *TP53INP1* combined with reduced presence of serum in the cell culture medium would alter the cells’ propensities to undergo apoptosis versus differentiate. We consequently measured the mRNA expression of pro-apoptotic markers BCL2-associated agonist of cell death (*BAD)* and BCL2-associated X (*BAX*), as well as the anti-apoptotic marker BCL2-like 1 (*BCL-X*) in *TP53INP1* siRNA-treated muscle cells. Interestingly, mRNA expression of *BAD* was unaltered whereas *BAX* expression was markedly upregulated after knock-down of *TP53INP1* with a simultaneous increase in *BCL-X* expression (Fig. 4A), thus indicating increases in both pro- and anti-apoptotic gene expression. Beyond regulating apoptosis, TP53INP1 has previously been reported to regulate the transcriptional activation of p53 target genes involved in myogenesis, including the cyclin-dependent kinase inhibitor 1 (p21)^33^. Interestingly, although *P53* expression was not affected by *TP53INP1* knock-down, p21 mRNA expression was robustly upregulated in *TP53INP1* siRNA-treated cells, while the expression of *RB1*, another p53-induced gene required for myogenesis^35,36^, was unaltered (Fig. 4B). Hence, despite lack of change in myogenic transcription factors, our data suggest that TP53INP1 may influence apoptosis and cell cycle exit during early differentiation, with a visible effect on myotube size.

## Discussion

In the present study we demonstrated reduced autophagy response to metabolic stress in human muscle precursor cells from humans with T2DM. Upon *in vitro* differentiation, these cells had lower protein levels of ATG7, up-regulated mRNA levels of *DRAM1* and down-regulated mRNA levels of *VAMP8*. In addition, *TP53INP1* was downregulated in the early differentiation state. Through a loss-of-function approach we demonstrated that this downregulation results in an increase in the apoptosis genes *BAX* and *BCL-X* and the cell cycle regulator *p21* at early differentiation, and enlarged myotubes once the cells are fully differentiated. Our data thus provide novel insight into the mechanisms manifested in muscle precursor cells in T2DM and highlight that a dysregulation of autophagy genes may contribute to the muscular phenotype of T2DM in humans.

The study was limited to *in vitro* experiments and thus only reflects the dysregulations in autophagy that are manifested in the muscle precursor cells even after *in vitro* expansion. However, an advantage is that we have utilized isolated cells from multiple human donors, thus including a biological variation from individuals that are suffering from T2DM. Moreover, using a cell-based approach allows us to study autophagy flux, which importantly cannot be done in muscle biopsies. In line with this, attempts to assess whether baseline autophagy is dysregulated in skeletal muscle biopsies in type 2 diabetes patients have resulted in conflicting findings, reporting either down-regulation^37^ or no difference from healthy controls^18^. These results might reflect the difficulties of measuring a dynamic mechanism such as autophagy in a biopsy including several cell types as well as muscle cells in different stages from precursor cell to differentiated myotube.

An increase in basal autophagy during C2C12 myogenesis has been previously demonstrated in several studies^4,9,38^; the current study provides the first evidence that this also occurs during human myogenesis. We previously showed that myogenesis is impaired in T2DM muscle cells, and that this dysregulation was associated with impaired signaling of p53 and the p53 target gene p21^19^. The current study support this concept of altered p53 signaling in T2DM muscle cells by demonstrating that two direct transcriptional targets of p53 (*DRAM1* and *TP53INP1*)^27,39^ are dysregulated in T2DM cells. While we expected to detect a difference in basal autophagy between cells from healthy and T2DM donors, we only found a difference in expression of ATG7, although this was not reproducible in our global gene expression analysis. ATG7 is a critical regulator of autophagy^40^; however, further studies are required to explore the potential dysregulation of autophagy in T2DM muscle cells. Nevertheless, as ATG7 has previously been shown to regulate the response to metabolic stress through interaction with p53 and subsequent induction of p21 transcription^24^, our finding that ATG7 is downregulated in T2DM muscle cells further supports the notion of altered p53 signaling in T2DM muscle cells.

Genome-wide association studies have identified *TP53INP1* as a diabetes susceptibility locus^26,41^ and interestingly, *tp53inp1*-deficient mice have chronic oxidative stress accompanied by insulin resistance and an increased number of dysfunctional mitochondria due to PINK1/ PARKIN-mediated impaired mitophagy^42^. We found that *TP53INP1* was downregulated in early differentiation, when cells were confluent but not yet fused. Muscle precursor cells committed to differentiation are prone to undergo apoptosis^43^ and TP53INP1 can promote apoptosis in an autophagy-dependent manner^34^. Paradoxically, we found that *TP53INP1* knockdown increased mRNA expression of both pro-and-antiapoptotic genes, while also increasing *p21* expression. This may be explained by previous studies showing that although TP53INP1 promotes apoptosis, TP53INP1 deficiency leads to ROS accumulation and consequentially upregulation of p21 and BAX expression^44^. In turn, p21 conveys anti-apoptotic effects to differentiating muscle cells^31^ and promotes BCL-X expression^45,46^; thus the observed increased expression of *BCL-X* after *TP53INP1* knock-down might reflect a compensatory p21-mediated anti-apoptotic response. Given that p21 is involved in cell cycle exit during early differentiation, this up-regulation might also explain our observation of enlarged myotubes following *TP53INP1* knockdown. Thus, despite the lack of alterations in the measured myogenic markers, we propose that the downregulation of *TP53INP1* in muscle precursor cells from people with T2DM might affect the early state of myogenesis leading to an imbalance between apoptosis and cell cycle exit, which may disturb proper differentiation. Nevertheless, our studies were limited to mRNA expression analysis; the use of more functional apoptosis assays would be necessary to explore the role of TP53INP1 in myogenesis-associated apoptosis. In line with this, further mechanistic studies examining exactly how TP53INP1 interacts with the autophagic machinery, such as co-localization experiments would be required to elucidate the specific roles of TP53INP1 in autophagy regulation.

Although we identified both *VAMP8* and *DRAM1* to be dysregulated in muscle cells derived from humans with T2DM, we were unable to further pinpoint their function in the current cell model. VAMP8 is a soluble N-ethylmaleimide-sensitive factor attachment protein receptor (SNARE) involved in autophagosome-lysosome fusion^47^, which has also been described in adipocyte GLUT4 trafficking^48–50^. Interestingly, *Vamp8* knock-out mice have markedly increased insulin sensitivity and muscle glucose uptake^51^, suggesting that the downregulation in human muscle cells from people with T2DM, might be a compensatory action to ameliorate impaired insulin action. This idea however remains to be explored. DRAM1 is required for p53-regulated autophagy and is upregulated in response to genotoxic stress^28^. The fact that we found *DRAM1* to be upregulated in fully differentiated T2DM myotubes may thus be a consequence of cellular stress in the T2DM cells, and it may explain why loss of *DRAM1* in undifferentiated muscle cells had no effect on myogenic progression.

## Conclusion

Our data suggest that muscle precursor cells from humans with T2DM have an altered expression of autophagy markers during metabolic stress and we identify a gene expression profile of autophagy-associated genes which are dysregulated in these cells. One of these genes, *TP53INP1*, seems to act in early myogenesis to regulate the balance between apoptosis and cell cycle exit and might thus be important *in vivo* to mediate proper muscle differentiation.

## Supplementary information

Supplementary data

## Data Availability

The data that support the findings of this study are available from the corresponding authors upon reasonable request.
