# Supplementary material for: Dysregulated autophagy in muscle precursor cells from humans with type 2 diabetes
Source: Sci Rep. 2019 Jun 3;9:8169. doi: 10.1038/s41598-019-44535-2 (PMC6546785; doi:10.1038/s41598-019-44535-2)
Supplement: Supplementary file 1 — Supplementary data [file 41598_2019_44535_MOESM1_ESM.pdf]

Dysregulated autophagy in muscle precursor cells from humans with type 2 diabetes

Henriksen TI*, Wigge LV, Nielsen J, Pedersen BK, Sandri M, Scheele C.

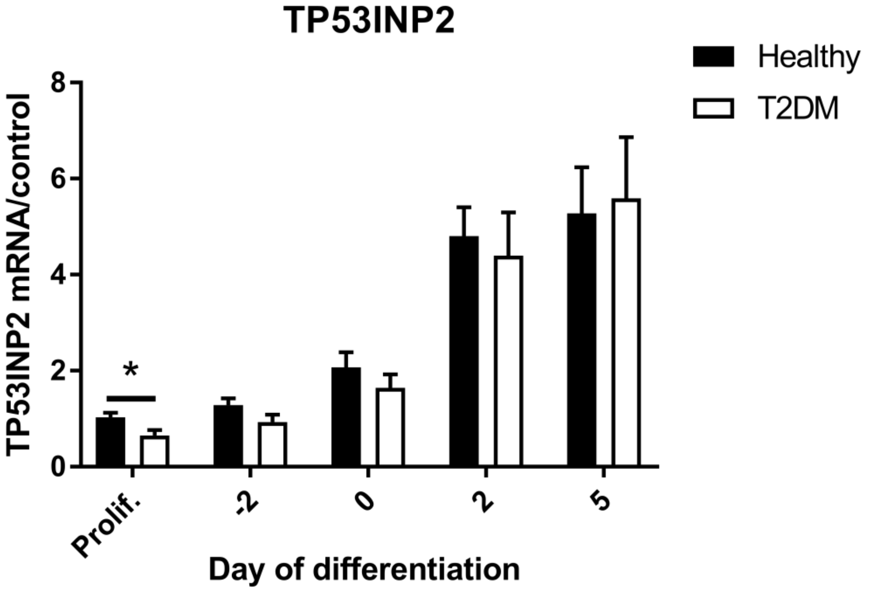

Figure S1. ***TP53INP2*** **expression in myoblasts derived from humans with T2DM and healthy control subjects.**
Myoblasts from healthy controls or T2DM donors were harvested under proliferation, at confluence (day -2), as aligned, undifferentiated myoblasts (day 0), at early differentiation, after 2 days in differentiation medium (day 2) and as fully differentiated moytubes, after 5 days in differentiation medium (day 5). *TP53INP2* mRNA was measured by RT-qPCR in healthy or T2DM muscle precursor cells. mRNA expression was normalized to the geometric mean of *18s*, *B2M* and *PPIA* mRNA. Data are means ± SEM. * indicates $P < 0.05$.

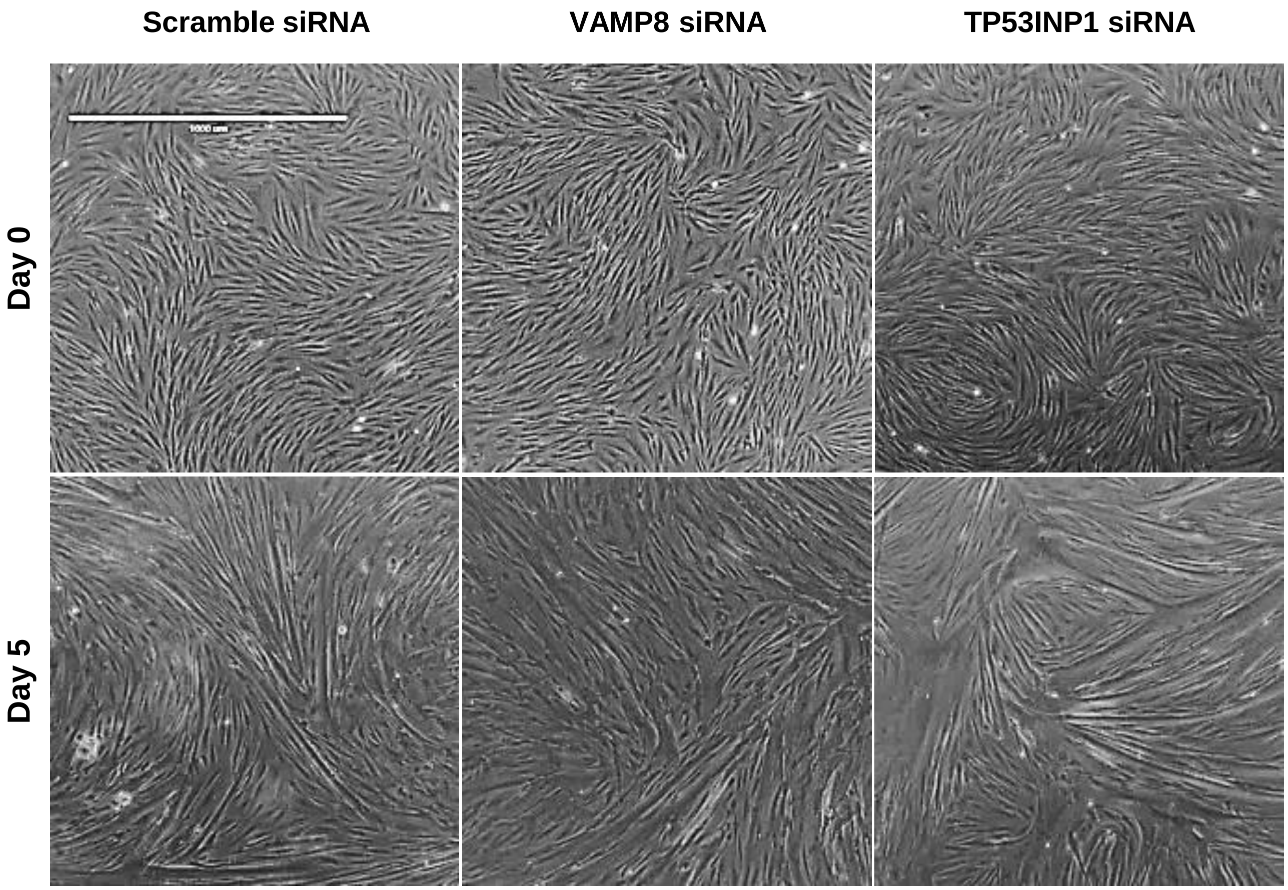

Figure S2. Effect of *VAMP8* and *TP53INP1* knock down on muscle cell morphology. Myoblasts from healthy controls (n=5) were treated with 20nM siRNA oligonucleotides targeting *VAMP8* or *TP53INP1*, or with a non-targeting scrambled control. Cells at >90% confluence were treated with siRNA and harvested 48 hours or 7 days after siRNA treatment; indicated as day 0 or day 5 respectively. Light microscopy images of siRNA-treated cells at 4X magnification; scale bar indicates 1000µM.

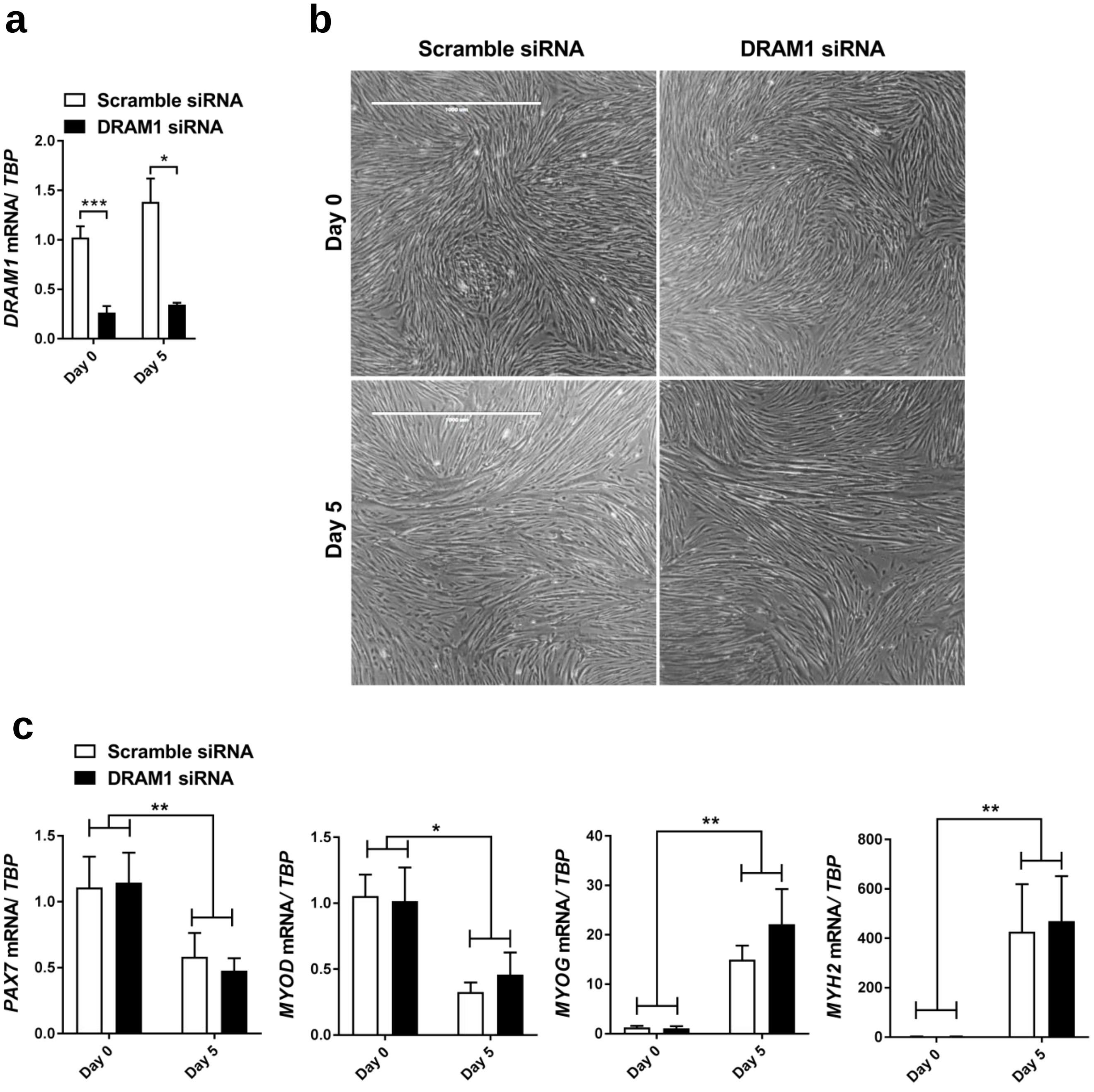

Figure S3. Effect of *DRAM1* knock down on myogenic marker expression.
Myoblasts from T2DM donors (n=5) were treated with 20nM siRNA oligonucleotides targeting *DRAM1*, or with a non-targeting scrambled control Cells at >90% confluence were treated with siRNA and harvested 48 hours or 7 days after siRNA treatment; indicated as day 0 or day 5 respectively. (**a**): *DRAM1* mRNA levels were measured by RT-qPCR and normalized to *TBP* mRNA. (**b**): Light microscopy images of siRNA-treated cells at 4X magnification; scale bar indicates 1000µM. (**c**) Expression of myogenic markers *PAX7*, *MYOD*, *MYOG* and *MYH2* in T2DM myoblasts treated with DRAM1 or control siRNA. Data are means ± SEM. * indicates $P < 0.05$; $^{**}P < 0.01$, $^{***}P < 0.001$.

a

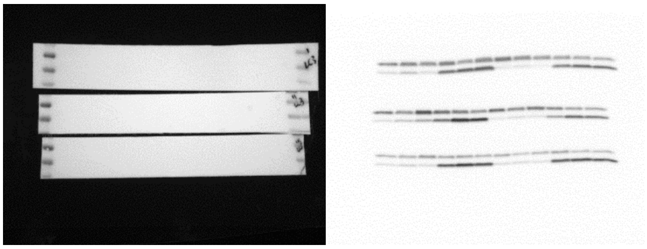

b

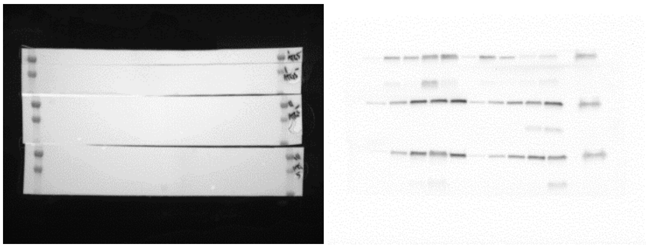

c

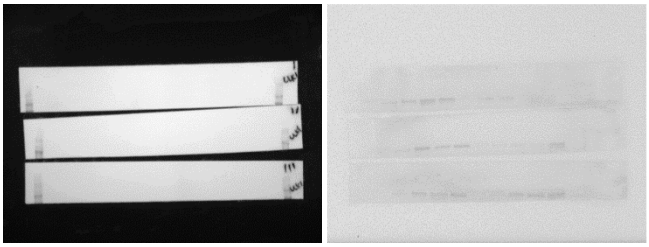

d

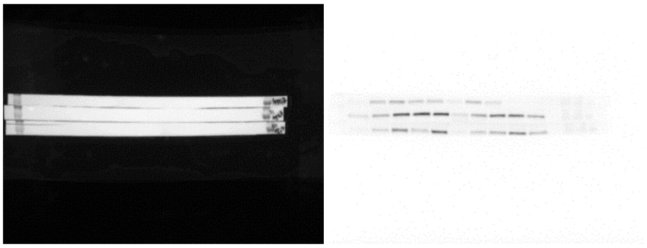

e

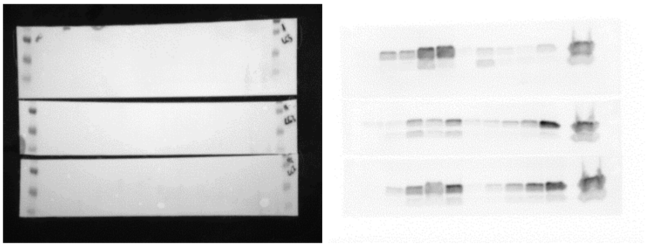

Figure S4. Unedited immunoblots. Protein ladder (left) and chemiluminescent images (right) of LC3II/I (**a**; figure 1 A), ATG5 (**b**), ULK1 (**c**), ATG7 (**d**) and LC3II/I (**e**; Figure 1 C).

Primer	Genomic locations (nt)	Targets
*B2M*F	319-338	*B2M*
*B2M*R	311-391	
*BAD*F	181-200	*BAD*
*BAD*R	726-703	
*BAX*F	165-185, 217-237, 312-332, 349-369, 364-384,	*BAX* transcript variants 1, alpha, beta, gamma, delta, zeta, lambda, sigma
BAXR	268-251, 320-303, 415-398, 452-435, 467-450,	
*BCLX*F	198-217, 428-447	*BCLX* transcript variants 1 and 2
*BCLX*R	270-247, 500-477	
*DRAM1*F	316-335	*DRAM1*
*DRAM1*R	421-402	
*MYH2*F	27-46, 69-88	*MYH2* transcript variants 1 and 2
*MYH2*R	118-98, 160-140	
*MYOD*F	786-803	*MYOD*
*MYOD*R	901-884	
*MYOG*F	456-473	*MYOG*
*MYOG*R	549-531	
*P21*F	410-431, 412-433, 465-486, 575-596, 617-638,	*P21* transcript variants 1-5
*P21*R	536-519, 538-521, 591-574, 701-684, 743-726	
*P53*F	919-938, 979-998, 1052-1071, 1299-1318, 1236-1255, 1239-1258, 1356-1375, 1372-1391,	*P53* transcript variants 1-8
*P53*R	1003-984, 1063-1044, 1136-1117, 1320-1301, 1323-1304, 1383-1364, 1456-1437, 1440-1421	
*PAX7*F	904-887, 744-727	*PAX7* transcript variants 1-3
*PAX7*R	826-843, 666-683	
*PPIA*F	10-29	*PPIA*
*PPIA*R	122-100	
*RB1*F	2807-2827	*RB1*
*RB1*R	2899-2877	
*TBP*F	237-260	*TBP*
*TBP*R	323-304	
*TP53INP1*F	395-416, 404-425	*TP53INP1* transcript variants 1 and 2
*TP53INP1*R	510-491, 519-500	
*VAMP8*F	216-237	*VAMP8*
*VAMP8*R	351-331	

Table S1. Genomic location and targets of RT-qPCR primers.
